# A standardised approach to quantifying activity in domestic dogs

**DOI:** 10.1098/rsos.240119

**Published:** 2024-07-17

**Authors:** Kamila Karimjee, Rachel C. M. Harron, Richard J. Piercy, Monica A. Daley

**Affiliations:** ^1^ Comparative Neuromuscular Diseases Laboratory, Department of Clinical Science and Services, Royal Veterinary College, London NW1 0TU, UK; ^2^ Structure and Motion Laboratory, Department of Comparative Biological Sciences, Royal Veterinary College, Hawkshead Lane, Hatfield AL9 7TA, UK; ^3^ Neuromechanics Laboratory, Department of Ecology and Evolutionary Biology, University of California, Irvine, CA 92697, USA

**Keywords:** accelerometer, cut-point, threshold, dog, monitoring, wearable

## Abstract

Objective assessment of activity via accelerometry can provide valuable insights into dog health and welfare. Common activity metrics involve using acceleration cut-points to group data into intensity categories and reporting the time spent in each category. Lack of consistency and transparency in cut-point derivation makes it difficult to compare findings between studies. We present an alternative metric for use in dogs: the acceleration threshold (as a fraction of standard gravity, 1 g = 9.81 m/s^2^) above which the animal's X most active minutes are accumulated (MX_ACC_) over a 24-hour period. We report M2_ACC,_ M30_ACC_ and M60_ACC_ data from a colony of healthy beagles (*n* = 6) aged 3–13 months. To ensure that reference values are applicable across a wider dog population, we incorporated labelled data from beagles and volunteer pet dogs (*n* = 16) of a variety of ages and breeds. The dogs' normal activity patterns were recorded at 200 Hz for 24 hours using collar-based Axivity-AX3 accelerometers. We calculated acceleration vector magnitude and MX_ACC_ metrics. Using labelled data from both beagles and pet dogs, we characterize the range of acceleration outputs exhibited enabling meaningful interpretation of MX_ACC_. These metrics will help standardize measurement of canine activity and serve as outcome measures for veterinary and translational research.

## 1. Introduction

Exercise is a key component of maintaining a dog's welfare. Increased levels of exercise reduce stress [1] and reduce the risk of developing obesity [2,3]. Exercise intolerance, or reduced spontaneous activity, can be early indicators of a wide variety of diseases [4,5], and suggest reduced welfare or pain [6,7]. Consequently, tracking changes in activity patterns might provide early detection of various health and welfare concerns. This is of particular interest to animal health professionals and pet owners wanting to take a proactive approach to prevention of disease. However, long-term changes in activity are difficult to detect via direct observation and could be subject to observer bias. Wearable, tri-axial accelerometers are commonly used to quantify activity and behaviour changes in animals [8,9] by measuring changes in direction and intensity of movement. Higher intensity movements are associated with higher levels of acceleration and deceleration.

Despite their growing popularity in quantifying activity, there is a lack of consensus on the appropriate way to process and interpret data from accelerometers. Commonly used outcome measures to summarize activity data can be abstract, population- or device-specific, and their derivation is sometimes unclear or undisclosed. Some studies have investigated inter-device variability [10,11], but beyond assessing correlations or absolute agreement between pairs of devices, often outputting different proprietary data units, the lack of transparency means it is not possible to draw further conclusions on how different processing parameters affect device data and, in turn, summary outcome metrics. Such approaches tend to involve aggregation of accelerometer data over time and dimensionless quantities are sometimes presented (e.g. ‘counts’ per unit time) without the accompanying information on their derivation. Importantly, metrics from many commercial grade devices are difficult to relate to locomotor behaviours of the species of interest [12–14]. Pre-processed data is then grouped into intensity categories (light, moderate, vigorous etc.) based on acceleration cut-points and reporting the time spent in each category. This type of approach also limits the information resolution about the intensity levels of activity within the cut-point categories.

An additional challenge of activity monitoring is that the choice of outcome measures and their measurement and processing parameters lack standardization across species, which limits their use in the veterinary setting [15,16]. Research in human-based activity monitoring has made use of accurate energy expenditure data to define the range of activity intensity levels [17,18]. The approach of grouping data into intensity categories guided by energy expenditure (e.g. light, moderate, vigorous) has been applied in dogs [19], despite the sparsity of equivalent energy expenditure data, which is much more challenging to collect in dogs. An additional challenge is the enormous intra-species variation in dogs in terms of body size and morphology among different breeds [20]. Body size and morphology have impacts on metabolism, movement, speed and locomotion patterns, and therefore accelerometry signal characteristics. This raises the key question of whether grouping accelerometry data into intensity levels is appropriate for activity monitoring in dogs.

Wearable activity monitors must balance demand for computational power and battery life with small size and weight [21]. As a result, minimizing sample frequency and reducing computational load are important considerations in activity tracking algorithms. Fine tuning of measurement and processing parameters are important additional considerations. The choice of sample frequency can dramatically affect battery life [22]. Therefore, it is important to determine the optimum sample frequency for a particular application by ensuring that it is high enough to capture the signal of interest while considering the trade-offs with longer battery life where appropriate. The frequencies exhibited by the signal of interest will vary between species and depend on the types of behaviours one wishes to capture. In terms of quantifying physical activity, considering the range of stride frequencies of the species of interest can inform this decision. Dogs have a stride frequency range of approximately 2–4 Hz across a range of gaits [23]. The Nyquist-Shannon theorem can then be applied, which states that the sample frequency must be at least twice the frequency of the signal you wish to capture in order to maintain integrity of the signal and prevent data loss [24,25]. We deduce, therefore, that the minimum sample frequency for capturing meaningful activity based on gait (beyond the simplified categories of ‘active’ versus ‘inactive’) in dogs should be 8 Hz.

To avoid the need to determine appropriate intensity cut-offs for a wide range of dog breeds and sizes, we propose the use of an alternative cut-point free approach to activity monitoring in dogs: we report the acceleration threshold (as a fraction of standard Earth gravity, 1 g = 9.81 m/s^2^) above which the dogs' X most active minutes are accumulated over a 24-hour period (MX_ACC_). This method was first outlined for use in humans by Rowlands *et al*. [26] as a ‘data-driven, meaningful outcome variable’ for tracking changes in activity in humans through wearable accelerometers. The values of X-minutes can be chosen according to a particular study requirement, with lower values of X-minutes characterizing higher intensity activity and higher values of X-minutes characterizing lower intensity activity. This method is particularly useful in a comparative context because it meaningfully translates among animals of different size. The original units of measurement are retained (*g:* fractional values of standard gravity) and have inherent meaning as a physical quantity: acceleration intensity. This allows for values to be compared across populations and differences in magnitude can be meaningfully compared and contrasted while maintaining interpretability.

The main objective of this study was to investigate the utility of the MX_ACC_ metrics in dogs. As part of demonstrating the robustness of this method, we have also outlined a data-driven approach to derive the most appropriate epoch length to process the dog data, and finally to quantify the impact of modifying epoch length and measurement sample frequency on the output MX_ACC_ data.

## 2. Material and methods

We used two cohorts in this exploration of the utility of MX_ACC_ metrics for canine activity monitoring. A labelled dataset from two cohorts of dogs was collected: a research Beagle colony and a cohort of volunteer pet dogs. Data were collected from both sources to enable comparison of values derived from the research colony with a more diverse (pet) dog population. This contributed towards establishing a set of reference values for specific behaviours that would be applicable across breeds and different settings.

### 3.1. Animals

#### 3.1.1. Dog colony

This study was conducted on animals maintained under a UK Animals (Scientific Procedures) Act 1986 (ASPA) project licence as part of a wider natural history study involving the beagle colony, however, this specific work was sub-threshold. Adult dogs were housed (12-hour light/dark cycle; 15–24°C) in large kennels in groups, until pregnant females were close to whelping when they were housed in single kennels. Puppies were weaned at 10–12-weeks old, then grouped in their litters until approximately 4 to 5 months of age. Adults were then housed in groups of 2–4 animals. Kennel sizes varied slightly but were within the range of 6.2–6.6 m^2^. Dogs were fed Burns (Burns Pet Nutrition Ltd, Wales, UK) puppy or adult feed, as required, twice a day ad lib, with daily human interaction and access to outdoor runs and grassy paddocks (approx. 100 m^2^) in groups of up to five dogs, typically between the hours of 8am–3pm. Husbandry conditions exceeded the minimum Animals (Scientific Procedures) Act 1986 (ASPA) requirements. This study included six wild-type (normal), male puppies born into the colony in early 2020 from three litters (table 1).

**Table 1.** Litter groupings and birth date details for each colony dog included in this study. The same litter code indicates that dogs were from the same litter.

dog code	litter code	birth date
DN583	AF	26/03/2020
EC166	AF	26/03/2020
BL886	AAG	11/04/2020
BT374	AH	21/04/2020
CS014	AH	21/04/2020
PE399	AH	21/04/2020

**Table 2.** List of relevant behaviours extracted from synchronised accelerometer and video recordings from both beagle colony and volunteer pet dogs, and corresponding active/inactive category.

behaviour	active/inactive
lie down	inactive
sit	inactive
stand	inactive
trot	active
walk	active

#### 3.1.2. Volunteer pet dogs

Additional data were collected from a separate group of animals to enable comparison of values derived from the research colony with a more diverse dog population and to establish a set of reference values that would be applicable across breeds. The pet dog portion of the study was approved by the Clinical Research and Ethical Review Board (CRERB) at the Royal Veterinary College (URN 2019 1901-2). All volunteer dogs (*n* = 16) were owned by staff or students at the Royal Veterinary College and the study was advertised via an internal message board. There were a total of nine male dogs and seven female dogs. The mean age was 4 years (± 3 years) and mean body mass was 19.2 kg (±10 kg). Further demographic details of age, breed and sex for individual dogs can be found in electronic supplementary materials S1 (electronic supplementary material Table S1).

### 3.2. Data collection

Data were collected via Axivity AX3 activity monitors (Axivity, UK). These have been previously validated for use in dogs [27–29] and their parameters are easily modifiable by the user through open-source software, unlike many other commercial devices. Devices were mounted onto each dog's neck collar using duct tape, and positioned ventrally to minimize likelihood of rotation of the logger over time. Additionally, the metrics selected were direction invariant to avoid any influence of device rotation. A sample frequency of 200 Hz was used for recording. This was a higher sample frequency in comparison to most industry standard devices (30–100 Hz [30–32]). Considering that one of the main objectives of this work was to quantify the effect of sample frequency on output metrics, we aimed to use a sample frequency that far exceeded that necessary to allow for meaningful comparisons to be carried out by downsampling to lower sample frequencies that are commonly used in industry devices. Using a higher sample rate also ensured that the loggers captured all high frequency activity and behaviour data.

#### 3.2.1. Dog colony

The loggers were mounted onto the dogs' collars before 12pm and removed after 12 pm, 2 days later. Dogs were habituated to wearing collars from 7 weeks of age. Dogs were sampled at monthly intervals between the ages of 3 and 18 months old and data were recorded for 48 h continuously. The dogs remained in their kennels during the period of monitoring and followed their normal daily routine. Paddock exercise was not provided during the monitoring period, however all kennels included an outdoor courtyard area which was always accessible. To develop aggregate metrics to represent activity over a 24-hour period, we split the 48-hour recording into 2× 24-hour segments. Each 24-hour segment was carried forward for analysis and calculation of aggregate metrics. The resulting metrics were then averaged across the 2× 24-hour periods per time point for each animal. Using an average of the output metrics from 2× 24-hour segments, we were able to establish a more representative measurement of that dog at a particular age point.

**Figure 1.**
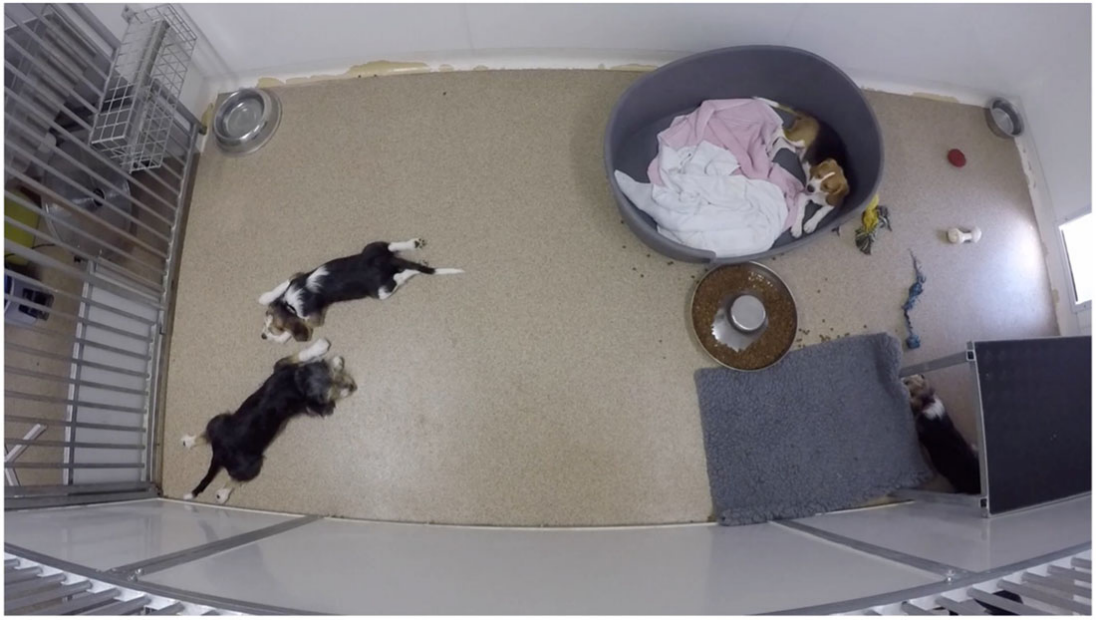
Example frame extracted from in-kennel video showing individuals CS014, EC166, BT734 and PE399.

In addition to the data described above, we also collected a smaller sample of video and accompanying accelerometer data (approx. 43 h across multiple recording sessions). The videos were collected at the end of the 48-hour segments within the kennel environment. Video data were recorded using GoPro Hero 5 black cameras and a gooseneck monopod with clamp attachment. Video was captured in 1080p and at 30 fps using the ‘Superview’ mode. This ensured that the whole kennel was visible with a single camera (see figure 1).

Upon starting recording, the collar was shown to the camera and visibly tapped 3× using a solid object (e.g. pen) in order to create spikes in the accelerometer data while within view of the camera. These spikes were used to synchronize the video and accelerometer data during labelling. The collars were then placed on the dogs and the start and end times were logged. The dogs were left undisturbed for a period of between 60 and 120 min.

#### 3.2.2. Volunteer pet dogs

We carried out 10-minute sessions of behavioural observation while the dog wore the AX3 logger. This observation period followed a protocol to encourage a range of behaviours in the dogs without specific commands (see electronic supplementary materials S2) to maintain an environment comparable to free-living. Dogs were put into a secure, enclosed pen [3 × 2.5 m] and were under video surveillance throughout the experiment. The video footage was live streamed to a screen outside the room to allow constant observation. Dog owners were able to watch the live footage and could decide to end the experiment early if they deemed their dog to be under significant stress due to being left alone in an unfamiliar environment. One experiment was terminated early due to anxiety and that dog's data has been excluded from the analysis.

### 3.4. Data processing

#### 3.4.1. Data labelling

All videos were manually synchronized with accelerometer data and labelled by three trained observers using open-source annotation software ELAN 6.0 (Max Planck Institute for Psycholinguistics, The Language Archive). A behavioural ethogram was developed (Supplementary Materials 4) and behaviours were labelled by trained observers according to the criteria described in electronic supplementary materials S4. Observers were instructed to label all segments of the recording, even if they did not include displays of behaviours of interest. Inter-observer reliability analyses were carried out to ensure consistency in labelling. All observers achieved >89% agreement (see electronic supplementary materials for further details). Segments where the dogs were not carrying behaviours of interest were labelled with ‘Transition’ if they were in view of the camera or ‘Out of frame’ if they were not within view.

#### 3.4.2. Active threshold derivation for epoch length analysis

We have explored the derivation of two key processing parameters: (1) derivation of an appropriate active/inactive threshold and (2) establishment of an appropriate epoch length for our dog data. However, each analysis required the use of both parameters. We chose to begin with the derivation of active/inactive threshold to quantify bouts of activity, which meant that an initial epoch length was required to process our data. The derived threshold was then taken forward and used to analyse potential epoch lengths. Lastly, we repeated the threshold derivation analysis with data processed using the newly established epoch length to confirm that the threshold remained appropriate. All signal processing was carried out using a combination of MATLAB R2021a (Mathworks) and Python 3.

The first step to determining the appropriate epoch length for use in our data was to determine an active/inactive acceleration threshold to quantify bouts of activity. This was completed using collected labelled reference datasets and is explained further below. We completed this analysis using the beagle data, as well as the pet dog data, to compare the outputs to a more diverse group of animals.

Labelled data were imported into MATLAB R2021a for processing. A 6^th^ order Butterworth band-pass filter was used in both directions (‘filtfilt’ function) to obtain zero phase lag, with cut-off frequencies of 0.28 Hz and 32.76 Hz (stop band: 0.1 Hz and 50 Hz, pass band: 0.5 Hz and 20 Hz). The filter settings were chosen to both remove the acceleration due to gravity and any background noise. We also anticipated that behaviours of interest would all occur at frequencies between the cut off frequencies listed (1). The labelled, filtered tri-axial data were used to compute the vector magnitude of acceleration, using the formula below:

$$\mathrm{vector}\;\mathrm{magnitude}=\;\sqrt{x^{2}+y^{2}+z^{2}}.$$

This allowed for the magnitude of the acceleration captured in each axis to be quantified, independent of direction. The labelled vector magnitude data were then split into 1 s epochs and each segment was assigned an ‘active’ or ‘inactive’ label, based on the groups shown in table 2. The mean was calculated for that segment. The 1 s epoch duration is commonly used in industry [30] and served as an initial starting point for derivation of activity threshold. In the cases where more than one label was present across a single 1 s segment, the label corresponding to >50% of the points was assigned to the whole segment. If there was no label that was assigned to greater than 50% of points, this segment was discarded and not taken forward for analysis.

**Figure 2.**
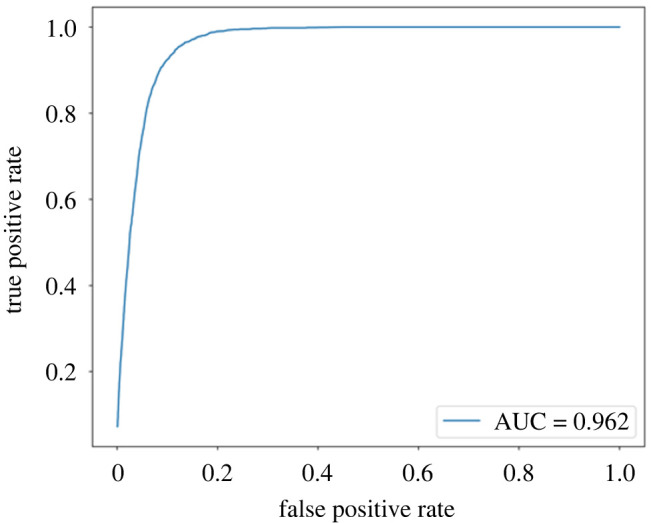
ROC curve showing changes in false positive and false negative rates with iterations of active/inactive threshold values using the beagle colony data.

The labelled data were then split into active and stationary behaviours (table 2) and exported to Python 3 for further analysis. Data were split into a training and test fractions (80:20) and a Receiver-operating characteristic (ROC) analysis was carried out. ROC curves visualize the trade-off between sensitivity and specificity for all possible cut-off thresholds for a test (figure 2), to enable selection of an optimum threshold that maximizes both sensitivity and specificity.

Sensitivity=True  Positive Rate

Specificity=1−False Positive Rate

The area under the curve (AUC) also provides a numerical measure of usefulness of a particular test. We iterated through thresholds between 0.001 and 0.5 g in increments of 0.001 g. We computed the differences between sensitivity and specificity values per threshold and selected the threshold with the minimum difference between the two quantities. The resulting threshold for the beagle data was 0.154 g, and for the pet dogs was 0.159 g.

We then applied the 0.154 and 0.159 g thresholds to the testing fraction of the labelled data from both dog populations to confirm suitability and compare differences. We also tested the thresholds between 0.154 and 0.159 g to provide extra context and observe high levels of sensitivity and specificity across both groups. However, the most balance between the statistics is observed at the derived optimum threshold for each group (0.154 g beagle colony, 0.159 g pets).

#### 3.4.3. Evaluating the effect of epoch length on activity measures

Once data are collected, smoothing by aggregating the data over a time window of fixed length, or ‘epoch’, is a tool that can be used to further reduce computational load while providing a more reliable estimate of the acceleration frequency. Epoch length refers to a time window of fixed length across which data points are aggregated during a recording. This is particularly helpful for devices with lower memory capacity, which is often the case in wearable devices due to their extremely small size. There is disagreement over the choice of appropriate epoch length [33]. Meta-analyses on specific age ranges of human data have examined the differences in data processed using different epoch lengths [34–36] but beyond advising shorter epoch lengths to capture shorter bursts of activity, no further guidance is available for quantitatively determining the appropriate length for a particular application.

Epochs are used for several reasons including reducing computational load while increasing reliability by averaging data points over a time period, versus simply reducing the sample frequency. The choice of epoch length should be a decision made in the context of the data collected. Given that we are investigating activity patterns, including counts and durations of bouts of activity, the choice epoch length was an important consideration. Here, we adopted a data-driven approach to explore how epoch length influenced the measured outcome variables.

To determine the appropriate epoch length for our dog data, we processed 24-hour activity data using different epoch lengths between 0.005 s and 1 s, in 0.005 s increments. The increment size was chosen as data were collected at 200 Hz, meaning that the changes were as granular as possible, incrementing by a single sample period each time. A 6^th^ order Butterworth band-pass filter was then used in both directions (‘filtfilt’ function) to obtain zero phase lag, with cut-off frequencies of 0.28 and 32.76 Hz (stop band: 0.1 and 50 Hz, pass band: 0.5 and 20 Hz). Using the epoched data, we calculated the maximum activity bout duration (greater than 0.154 g or greater than 0.159 g, derivations described above) over the 24-hour period.

**Figure 3.**
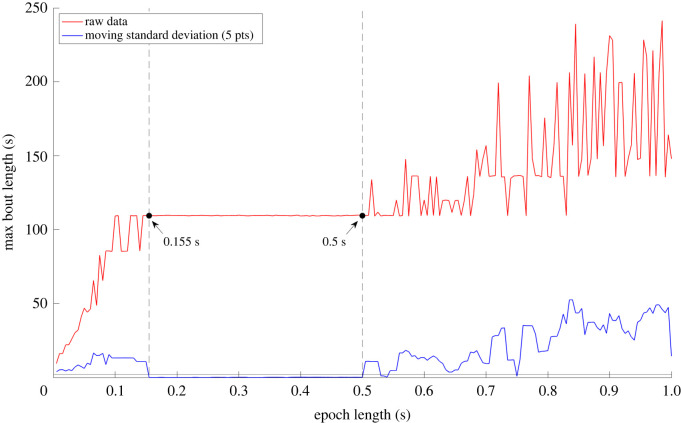
An example of the effect of increasing epoch length in increments of 0.005 s between 0 and 1 s on maximum active bout duration in seconds (red) for one example dog, PE399 at 8 months old. The activity threshold was 0.154 g. The moving standard deviation is also shown (blue). Dotted lines represent the start and end of the portion of the maximum active bout duration curve falls below a standard deviation of 2 s. The corresponding epoch lengths at the start and end of the plateau are labelled (0.155 s and 0.5 s). The optimum epoch length was determined by taking the mean of the start and end points of the plateau which was 0.3 s, effectively corresponding to a filter of 3.33 Hz which falls within the reported range of stride frequencies in dogs (approx. 1 to 5 s^−1^ dependent on body size and movement speed) [23].

We carried out this analysis twice to evaluate both thresholds derived from the pet dog and beagle colony labelled data. The maximum bout durations were then plotted against epoch length for each 24-hour recording (figure 3). The resulting graph showed a rapid increase in maximum bout duration as epoch length increased, up to a certain point. The graph then plateaued before showing further changes, which we infer relates to epoch lengths that best captures overall activity levels. It follows that the graph would then start to increase again at a certain point, as distinct activity bouts may then coalesce. Using this plotted curve, we aimed to capture the midpoint of the first plateau without any manual input. To do this, alongside the maximum active bout duration, a moving standard deviation (5 points) was also plotted and the first point where the standard deviation fell below 2 s, and remained below for at least five data points, was deemed the start of the plateau. The end was marked by the next point that the standard deviation increased above 2 s (figure 3). We then computed the mean of the two epoch lengths corresponding to the start and end of the plateau. This provided us with a suitable epoch length per individual 24-hour recording. We carried out this analysis for the 1^st^ 24-hour period for each monthly recording per dog in the beagle cohort. This was repeated using both active/inactive thresholds [0.154 and 0.159 g] to assess the impact of varying the threshold on the optimum derived epoch length. Lastly, we computed the overall mean epoch length of these values. The resulting epoch value using the pet dog active/inactive threshold (0.159 g) was 0.3 s and the beagle colony threshold (0.154 g) was also 0.3 s. We have chosen to take this forward as the most appropriate epoch for processing canine activity data. For the purposes of this work, we will be using the active/inactive threshold derived from the beagle colony for further analyses.

During our active/inactive threshold derivation, we described using a 1 s epoch length as a starting point to determine the active/inactive threshold of 0.154 g. This was due to the requirement of needing an active threshold to perform the epoch analysis. To confirm that 0.154 g is still an appropriate threshold for use with our newly derived epoch length of 0.3 s, we re-segmented our labelled beagle colony data into segments, using the same criteria described above. We then applied the active threshold to the data to classify each segment as active or inactive, resulting in the classification scores seen in table 3. The high levels of sensitivity (86.6%) and specificity (91.1%) confirmed that the active threshold of 0.154 g remained appropriate for the data.

**Table 3.** * denotes optimum threshold derived from pet dog population, ** denotes the optimum threshold derived from the beagle colony. Evaluation results of optimum thresholds to quantify active/inactive behaviour derived from the pet dog (0.159 g) and beagle colony (0.154 g) data to maximise and maintain balance in both sensitivity and specificity (i.e. minimise the differences between the two values) in each dog population. Both thresholds were tested on 20% test fractions of each dataset and the results are shown above.

active/inactive threshold (g)	pet dogs		beagle colony	
	*Sens. (%)*	*Spec. (%)*	*Sens. (%)*	*Spec. (%)*
***0***.159*	89.3	88.9	89.6	91.3
***0***.***158***	89.3	88.9	89.6	91.3
***0***.***157***	89.5	88.8	89.8	91.2
***0***.***156***	90.0	88.6	90.1	91.0
***0***.***155***	90.2	88.5	90.4	90.6
***0***.154**	90.3	88.4	90.6	90.8

#### 3.4.4. Derivation of MX_ACC_ metrics

We report an alternative metric, first presented in humans by Rowlands and colleagues [26], for activity monitoring in dogs: the acceleration threshold (*g*) above which the animal's X most active minutes are accumulated (MX_ACC_) over a 24-hour time period. Examining the threshold above which the dog's X most active minutes are accumulated over a 24-hour period provides an understanding of the varying intensity levels of movement that are exhibited over a longer time frame, and how individuals compare to each other. The values reported are directly comparable between individuals and, importantly, are translatable between populations. The values of X used can be adjusted to suit the data that is being examined, with smaller values reflecting higher intensity activities and larger values reflecting lower intensity activities.

In this case study, we present MX_ACC_ data computed using X-minute values of 2, 30 and 60, based on previous recommendations for human use [26]. To calculate these data points, the epoched vector magnitude was sorted in descending order and the appropriate X-minute increment was indexed. The vector magnitude value at the X time index in the sorted data is the MX_ACC_ statistic.

#### 3.4.5. Analysis of dog-specific behaviours

Using labelled and segmented behaviours from the beagle colony, we have computed relevant reference values for mean acceleration output (*g*) during segments of specific behaviours. Behaviours and/or postures that were deemed most relevant for contextualizing activity intensity levels are shown in table 4.

**Table 4.** Relevant species-specific behaviours and associated mean and standard deviation of vector magnitude across segments. n denotes the number of 0.3 s segments labelled per behaviour.

behaviour	mean vector magnitude (g)	standard deviation vector magnitude (g)	n
lie down	0.02	0.05	212 994
sit	0.08	0.08	34 227
stand	0.13	0.09	42 718
trot	0.59	0.24	1486
walk	0.26	0.14	42 198

#### 3.4.6. Effect of modifying sample frequency and epoch length on aggregate metrics

Commercially available sensors often use measurement and processing parameters that are not modifiable by the end-user [10–12]. This can result in inconsistencies of these parameters across studies rendering them incomparable. To objectively address this problem, we quantified the impact of modifying sample frequency, epoch length and the interaction between them on our MX_ACC_ aggregate measurements characterizing 24-hour activity.

The 24-hour tri-axial accelerometer data was imported into MATLAB R2021a (Mathworks) and downsampled to a range of sample frequencies (‘interp1’ function using cubic interpolation). Once downsampled, the data were filtered to remove gravity using a low-pass Butterworth filter in both directions. The settings were chosen with the criteria of choosing the lowest order filter that loses no more than 0.01 dB in the passband (0.5 Hz) and has at least 30 dB of attenuation in the stopband (0.1 Hz). The settings varied slightly between recordings because they were dependent on the sample frequency of the signal, which was deliberately altered. The data were smoothed using a range of epoch lengths within the range of 0.3 s to 60 s (0.3, 1, 5, 10, 15, 30, 60), and the aggregate MX_ACC_ metrics were calculated. This was done for all 24-hour activity recordings for all individual animals.

We then imported the aggregate metrics calculated from the downsampled and smoothed data to SPSS Statistics (IBM SPSS Statistics 25) for analysis. Analyses using the interclass correlation coefficient (ICC) were carried out to assess reliability of output data computed using varying epoch lengths (0.3 s, 1 s, 5, 10 s). The same analyses were computed to assess reliability of output data at varying sample frequencies (200 Hz, 100 Hz, 50 Hz, 25 Hz, 10 Hz, 9 Hz, 8 Hz, 7 Hz, 6 Hz, 5 Hz). ICC estimates and their 95% confident intervals were calculated based on a single measure, absolute-agreement, 2-way mixed-effects model and were computed separately for sample frequency and epoch length. ICC examined the absolute agreement between varying parameters, however, we also wished to investigate the effects of combinations of varying sample frequency and epoch lengths on individual recordings and examine the interaction effect, if any, between them. A linear mixed effects model was used with sample frequency and epoch length as fixed effects and a random effect was added to account for variation between with individual dogs. Models were examined in order of complexity and goodness of fit was evaluated using Akaike's Information Criterion (AIC). F-statistics were computed and reported per fixed effect in each model.

## 4. Results

### 4.1. 24-hour MX_ACC_ data

**Figure 4.**
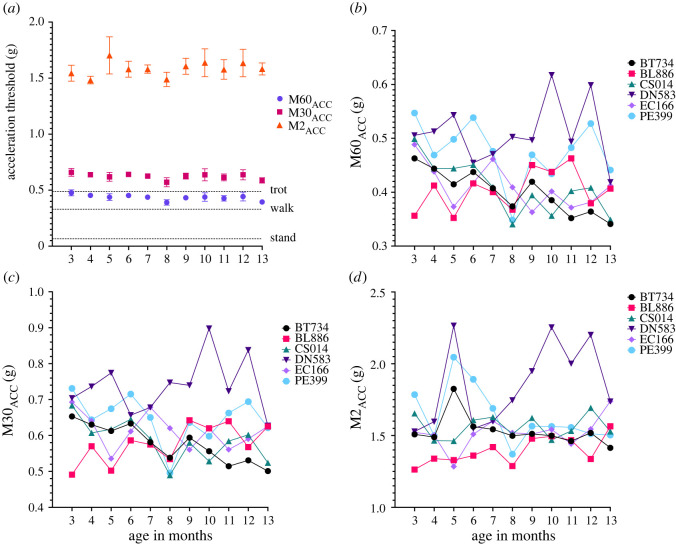
(*a*) MX_ACC_ data (mean and s.e.) from 6 WT dogs sampled at monthly intervals (3–13mo), (*b*–*d*) M60_ACC,_ M30_ACC_ and M2_ACC_ data respectively from individual dogs shown over time. The effect of age was not statistically significant (*p* > 0.05). Reference behaviours shown in 4a) were computed from labelled dog data.

We have computed M2_ACC,_ M30_ACC_ and M60_ACC_ values over a 24-hour period for six beagle colony dogs at monthly intervals between the ages of 3 and 13 months. Using a labelled pet dog dataset, we have contextualised the MX_ACC_ data with species-specific behaviours to contribute towards meaningful interpretation of the activity data. Mean MX_ACC_ measures showed consistent patterns across all ages sampled. Linear mixed effects analyses did not find age to be a significant effect for any MX_ACC_ metric (all *p* > 0.05). When considering the individual animals across the sampled age points, there was variation in MX_ACC_ values over time (figure 4*b–d*). The greatest standard error occurs ages 10 and 12 months old in all metrics, however this might be skewed by a single individual, DN583, who showed extremely high values at those ages. All dogs at all ages carried out greater than 60 min of activity at intensity levels greater than walking over a 24-hour period (figure 4*a*), but less than or equal to trotting, except for DN583 for whom the M60_ACC_ was over the trotting intensity level at 12 months old. All dogs carried out at least 30 min of activity at intensity levels approximately equal to trotting over a 24-hour period (figure 4*a*), with some individuals falling slightly above or below the trotting intensity value (0.591 g).

### 4.2. Balancing the effects of sample frequency and epoch length

#### 4.2.1. Sample frequency

**Figure 5.**
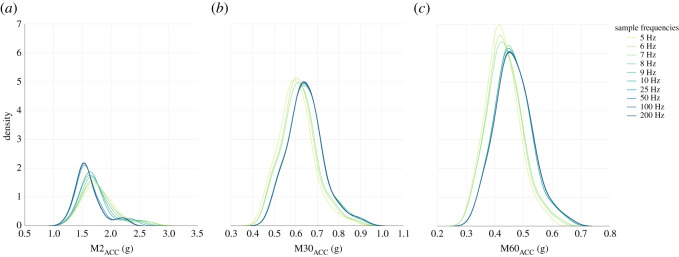
Distributions of M2_ACC_, M30_ACC_ and M60_ACC_ respectively, computed using data resampled to varying sample frequencies (5–200 Hz). Epoch length of 0.3 s was used for all frequencies. Intra-class correlation co-efficient agreement analyses showed strong correlations for all metrics.

We computed a collection of aggregate activity metrics at a range of sample frequencies and epoch lengths and assessed the impact of these variables on the outcome measures (table 5). Frequency density plots (figure 5) showed a shift towards higher values of M30_ACC_ and M60_ACC_ as sample frequency is decreased. M2_ACC_ values, however, showed the opposite when compared to M30_ACC_ and M60_ACC_ and there is a rightward shift in the distribution which increased the mean as the sample frequency decreased. The changes were most pronounced at frequencies of 10 Hz and below, with small differences in distribution for sample frequencies above 10 Hz. The observed differences across all MX_ACC_ metrics were supported by intra-class correlation coefficient analyses (all *p* < 0.0001), with M30_ACC_ agreement having a slightly lower single measures coefficient of 0.88 (0.75–0.93) when compared M2_ACC_ and M60_ACC_ metrics with higher coefficients at 0.94 (0.83–0.97) and 0.963 (0.89–0.98) respectively. Despite visual differences in the distributions, the high intra-class correlation coefficients suggested strong agreement in MX_ACC_ between varying sample frequencies when computed with an epoch length of 0.3 s.

**Table 5.** Linear mixed effects model output examining the effect of modifying sample frequency and epoch length on output metrics. * denotes effects are statistically significant (*p* < 0.001).

MX_ACC_ metric	epoch length		sample frequency		epoch length * sample frequency	
	*F* statistic	*p* value	*F* statistic	*p* value	*F* statistic	*p* value
**M2_ACC_**	2940.063*	<0.0001	3.553*	0.0002	4.487*	<0.0001
**M30_ACC_**	391.890*	<0.0001	25.640*	<0.0001	0.089	1.000
**M60_ACC_**	128.110*	<0.0001	31.954	<0.0001	0.081	1.000

#### 4.2.2. Epoch length

**Figure 6.**
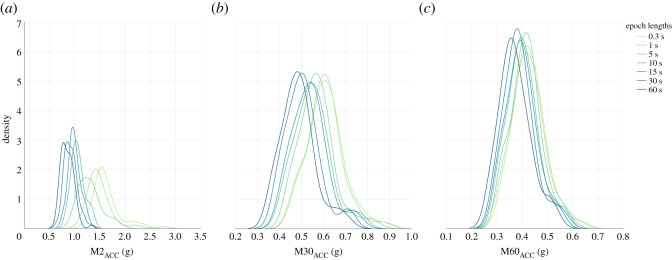
Distributions of M2_ACC_, M30_ACC_ and M60_ACC_ respectively, computed at varying epoch lengths (0.3–60 s). Sample frequency was unchanged from the original measurement frequency of 200 Hz. Intra-class correlation coefficient agreement analyses showed low agreement for M2_ACC_ (0.178), with stronger agreement for M30_ACC_ and M60_ACC_ (0.712 and 0.867 respectively).

**Figure 7.**
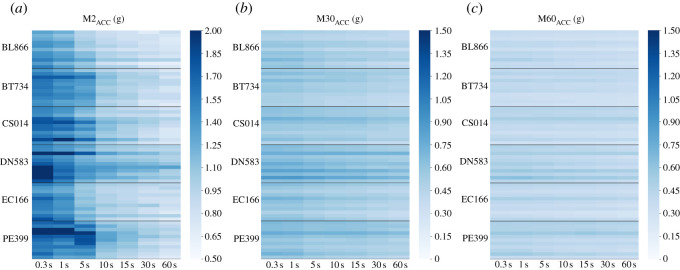
Heatmaps showing changes in individual M2_ACC_, M30_ACC_ and M60_ACC_ data when computed at varying epoch lengths (0.3–60 s). Sample frequency was unchanged from the original measurement frequency of 200 Hz. Each colour rectangle represents a single data-point from a single age-point per dog.

Epoch agreement density plots (figure 6) showed a clear shift towards lower values of MX_ACC_ data at all values of X as epoch length is increased. The differences were more marked for M2_ACC_ values when compared to M30_ACC_ and M60_ACC_. The difference is supported by ICC analyses (all *p* < 0.0001), with M2_ACC_ single measures coefficient of 0.18 (0.05–0.34). M30_ACC_ and M60_ACC_ both had higher coefficients at 0.71 (0.39–0.86) and 0.87 (0.70–0.93) respectively. To further explore these agreement values, the heatmap (figure 7) showed the differences in individual recordings processed with different epoch lengths for all MX_ACC_ metrics. In line with the low agreement coefficient, the M2_ACC_ values, quantifying the highest intensity levels, were most impacted by increasing epoch lengths. The differences were particularly marked in dogs with the highest M2_ACC_ values (DN583 and PE399). In line with the higher coefficients seen in the ICC analysis, indicating stronger agreement, M30_ACC_ and M60_ACC_ metrics remained more consistent with increasing epoch lengths.

#### 4.2.3. Sample frequency and epoch length in combination

The linear mixed effects model (LMM) revealed that both fixed effects, sample frequency and epoch length, were statistically significant. This was true for all MX_ACC_ metrics (*p* < 0.001). A significant interaction effect was also found between sample frequency and epoch length (*p* < 0.001) in the M2_ACC_ metric only. Epoch length had the greatest effect size for all MX_ACC_ metrics, with the largest observed in the M2_ACC_ data (*F* = 2940.06) when compared to sample frequency (*F* = 3.55). An increase in epoch length at all sample frequencies caused a decreased in MX_ACC_ metrics. An increase in sample frequency between 5 and 8 Hz resulted in an increase in M30_ACC_ and M60_ACC_ metrics at all epoch lengths, with the greatest increase computed between 7 and 8 Hz. This is unlike the M2_ACC_ metric, where mean M2_ACC_ values decreased as sample frequency increased up to 25 Hz for epochs 0.3 and 1 s. This pattern contrasts with all larger epochs tested, in which the mean M2_ACC_ values increased slightly with increasing sample frequency between 5 and 8 Hz before plateauing.

## 4. Discussion

We have outlined an approach to quantify long-term changes in activity in dogs using collar-based accelerometers. Firstly, we provided a case study of six normal research beagles sampled at monthly intervals between the ages of 3 and 13 months using MX_ACC_ metrics, first presented for use in humans [37], to characterize their movement patterns. Secondly, we provided a labelled reference dataset using a sample of volunteer pet dogs and used this to contextualize the metrics with relevant, species-specific behaviours, to support meaningful interpretation of movement intensity in dogs. Using these data, we characterised changes in long-term activity in a sample of research beagle colony dogs, then continue to derive the most appropriate active/inactive acceleration threshold. We completed this analysis using data from our beagle colony cohort, but also included a sample of pet dogs, from a shorter measurement period, to compare results and note any differences. We then outlined a data-driven method to derive the optimum epoch length, a key processing parameter in computing aggregate activity data. This approach incorporated the active/inactive threshold results, and was completed using the beagle colony result, then repeated with the pet dog result to demonstrate translatability to a more varied dog population. Lastly, we quantified the impact of modifying the epoch length and measurement sample frequency on the output aggregate data.

When searching for guidance on appropriate epoch selection, advice was qualitative and limited to advising caution when cross-comparing studies using different epochs [36]. There were no quantitative derivation methods or reasoning available and, given the known effects of modification of epoch lengths on under/over reporting aggregate measures [38–41], we felt it was important to develop a data-driven approach to derive the appropriate epoch length for this dog data that was robust and translatable to other studies. Incorporating the use of epochs during signal processing effectively adds an additional ‘notch’ filter that averages (and therefore smooths) signals at a specific frequency. Our results suggest an optimum epoch length of 0.3 s for monitoring activity levels in dogs, effectively corresponding to a filter of 3.33 Hz. Incidentally, this frequency falls within the range of stride frequencies (1 to 5 Hz) observed over a range of speeds and gaits in dogs of a range of body masses [23]. Filtering of frequencies at 3.33 Hz likely will remove small movements that occur around this frequency (e.g. single limb movement) that are not relevant to whole body activity, and so will rightly not be detected as indicating the start or stop of bouts of locomotion.

We collected labelled data from a group of volunteer pet dogs from a variety of ages, breeds and sexes to compare the resulting metrics to our more uniform sample of research beagles. We found similar optimal epoch length and active/inactive threshold between the pet dogs and beagle colony: epoch of 0.3 s, activity threshold of 0.59 g for pet dogs and 0.154 g for beagles, respectively. This likely reflects that acceleration values are dimensionless once normalized to fractional values of gravitational acceleration, and therefore largely size independent when comparing similar movement behaviours.

There are likely to be size-related effects on acceleration based activity metrics among breeds due to differences in locomotor behaviours among breeds of different morphology and body size; however, our sample size did not have sufficient numbers to systematically address the effects of breed and body size in the current analyses. Further work should include gathering larger samples of data from a wider variety of ages, breeds, sexes and sizes with specific recruitment targets for each category.

We assessed the impact of decreasing sample frequency on MX_ACC_ metrics to quantify the change in output data, but also to determine the most appropriate sample frequency to choose when monitoring dogs. At M30_ACC_ and M60_ACC,_ we observed an overall decrease in the data with decreasing sample frequency. This was expected, as these metrics were capturing lower levels of acceleration and so reducing the sample frequency may result in lower detection of activity because of less frequent sampling or missed short bouts. However, in the M2_ACC_ data, quantifying much higher intensity levels of activity, we observed the opposite. The shift in data was most apparent at frequencies below 25 Hz, suggesting that high intensity levels of activity might be more susceptible to curve smoothing when sampled at a lower frequency. It is also possible that there is added impact because of the high pass filter used to remove the gravity signal, as the decreasing sample frequency tended towards the cut-off frequency used. It is challenging to quantify the extent of this effect; however, it highlights the importance of considering the choice of sample frequency when collecting activity data and maintaining consistency between recordings. There is also the question of whether loss of important signal features starts to occur at around this signal level. We have previously examined the signal characteristics of a much smaller sample of dog activity data and recommended a minimum sample frequency of 25 Hz for use in dogs [42], this is supported by our new findings in this work. This is also in line with sample frequencies used by commercial devices in humans [43].

We found the MX_ACC_ to be a robust metric to quantify activity levels over a broad range of age in beagle colony dogs. This approach is not limited to dogs and can also be applied to larger-scale health surveillance in both clinical and research settings, as well as development of and adherence to exercise guidelines, with the potential to increase animal health and welfare. The advantages of these metrics include the ability to compare outputs with relevant behaviours and retain meaningful interpretation across populations. For example, if quantifying activity over a 24-hour period, it is understood that any individual would have two minutes that are objectively ‘the most active’ across the recording period, regardless of the intensity of those two minutes. Therefore, the most intensely active 2 min, M2_ACC_, for a dog could be directly compared with other dogs as well as other species: the difference in magnitude would be meaningful without requiring manipulation.

Physical activity is used as a marker for sensorimotor health and quality of life in both human and animal health [4,5]. Many health conditions use physical activity as a marker of disease progression, in both research and clinical settings [44–47]. Physical activity intervention programmes are also used for disease prevention in conditions such as dementia and generalized cognitive decline [48,49]. Robust, outcome measures are needed to quantitively assess changes in activity to provide standardization and ensure comparisons between studies. MX_ACC_ metrics would fulfil this need, facilitating direct comparisons among, and between, conditions, disease models and even species. The MX_ACC_ metrics also can support monitoring adherence to exercise guidelines (149). As demonstrated by Rowlands and colleagues in humans, this could also be very useful in dogs. Monitoring and adherence to exercise guidelines is challenging in dogs, especially in research settings where exercise standards are closely monitored by external bodies for regulatory and welfare purposes. For example, a target of 30 mins per day of exercise equivalent to trotting could be tracked using the M30_ACC_ metric combined with reference behaviours. The use of the M30_ACC_ here would enable objective assessment as to whether individual dogs have met this target and importantly, if not, quantify exactly how far away they were from meeting it.

We examined the effect of varying epoch lengths on our MX_ACC_ data and found that the greatest impact on the output data was with M2_ACC_ data (the highest intensity metric). This was expected as higher intensity activities tend to occur in shorter bursts. As such, increasing the epoch length results in an average taken across a longer time frame, possibly longer than the bout of activity itself, reducing the measured acceleration before the aggregate metric is computed. This is in line with findings from other work assessing changes in epoch length on cut-point approaches in children [38,39]. We also observed statistically significant decreases in M30_ACC_ and M60_ACC_ with increasing epoch length, however these were less marked compared to the M2_ACC_ values.

## 5. Conclusion

We have proposed an approach to long-term activity monitoring in dogs using a combination of MX_ACC_ metrics and species-specific reference activities and quantified the effect of modifying key measurement and processing parameters on aggregate data. Our data reveal that keeping parameters consistent is essential to enable meaningful comparisons between studies. Incorporating MX_ACC_ metrics into activity monitoring analyses will further standardize measurement of canine activity. Use of the reference activity levels derived in this work can be applied to future studies in combination with computed MX_ACC_ values to enhance interpretability of results. This will inform both development and adherence monitoring of exercise guidelines for dogs as well as serve as functional outcome measures for research and clinical applications.

## Data Availability

The dataset and relevant code supporting this article have been uploaded to Datadryad doi:10.5061/dryad.m0cfxppbs [50]. Supplementary material is available online [51].
